# Design and validation of an integrated meta-instrument for nursing assessment in home hospitalization: A study protocol

**DOI:** 10.1371/journal.pone.0351126

**Published:** 2026-06-23

**Authors:** Paula Barrué-García, Irene Llagostera-Reverter, Águeda Cervera-Gasch, Aurora Esteve-Clavero, María Jesús Valero-Chillerón, Víctor Ortiz-Mallasén, Marta Aquilué-Ballarín, David Luna-Aleixos, María Sánchez-Galán, Víctor M. González Chordá, on behalf of the VALENF-HAD Group

**Affiliations:** 1 Nursing Research Group (GIENF-241), Nursing Department, Universitat Jaume I. Castelló, Castelló de la Plana, Spain; 2 Consorcio Hospitalario Provincial de Castellón, Castellón de la Plana, Castellón, Spain; 3 Joint Research Unit NURSIA (“NURSing Care, Information Systems, Tecnology and Quality”) FISABIO-UJI, Valencia, Spain; 4 Hospital General Universitario de Castellón (FISABIO), Castellón de la Plana, Castellón, Spain; 5 Hospital Universitario Comarcal de Vinaròs (FISABIO), Vinaròs, Castellón, Spain; 6 Hospital Universitario de La Plana (FISABIO), Vila-Real, Castellón, Spain; 7 Nursing and Healthcare Research Unit (INVESTEN-ISCIII), Institute of Health Carlos III, Madrid, Spain; University of Hafr Al-Batin, SAUDI ARABIA

## Abstract

**Background:**

Hospital-at-home (HaH) programs provide an effective and safe alternative to conventional hospitalization, particularly for patients with multimorbidity or complex chronic conditions. In this setting, nursing assessment plays a key role, given the intermittent nature of care and the limited presence of other professionals. However, nursing documentation in HaH remains challenging because of organizational heterogeneity, fragmented records, and the coexistence of multiple assessment tools that create redundancy and compromise information quality. The overall objective of this study is to develop and validate a meta-instrument (VALENF-HAD) that integrates the assessment of functional capacity, pressure injury risk, fall risk, frailty, nutritional status, and sleep quality in patients admitted to hospital-at-home units on the basis of the analysis of other validated measurement instruments used in nursing assessment.

**Methods:**

A multicentre, cross-sectional study will be conducted in four public hospitals in Castellón Province, Spain. Participants will include adults (≥18 years) admitted to HaH units. Sociodemographic and care-related variables, along with risk-related variables, will be collected using the following instruments: functional capacity (Barthel Index and Lawton & Brody Scale), risk of pressure injuries (Braden Scale), risk of falls (STRATIFY Scale), frailty (FRAIL questionnaire), nutritional status (Nutritional Screening Initiative tool), and sleep quality (Athens Insomnia Scale). The development process of the meta-instrument consists of four phases: (I) analysis of instruments, constructs, and items; (II) meta-instrument design; (III) initial validation; and (IV) determination of clinimetric properties.

**Discussion:**

This study is expected to produce a parsimonious and specific meta-instrument for hospital-at-home settings that is capable of integrating functional, nutritional, sleep, fall risk, frailty, and pressure injury assessments. Its implementation will enhance the quality and standardization of nursing documentation, reduce administrative workload, facilitate early risk detection, and free up time for direct patient care. Ultimately, this study will contribute to advancing evidence-based nursing practices in home hospitalization.

## Background

Hospital-at-home (HaH) care represents an efficient alternative to conventional inpatient care for numerous medical and surgical conditions [1]. It is defined as a hospital-level care modality delivered in the patient’s home providing active monitoring and treatment for the patients who would otherswise require acute hospital care. Fro conceptual perspective, HaH encompasses two main operational models: admission avoidance (preventing hospitalization) and early discharge (shortening hospital stay), when patients do not require hospital facilities but still need active monitoring [2]. Both are aimed at reducing costs and complications associated with the hospital environment [3,4]. In Spain, HaH was implemented in 1981, although its development has been uneven and limited by the lack of a regulatory framework and the heterogeneity of organisational models [1].

Nursing care at home differs substantially from hospital-based care, as it is intermittent and often delivered in the absence of other health care professionals [5]. These circumstances increase nurses’ responsibility in clinical decision-making and require high-quality assessments to ensure effective interprofessional communication [6]. Moreover, home care constitutes one of the main settings for managing patients with complex chronic multimorbidity, characterised by heterogeneous needs that range from low-intensity care to high-dependency, complex clinical support [7]. Central to this model is a biopsychosocial approach, where social and family dynamics are prioritized alongside clinical stability. This framework fosters shared responsibility, integrating patient autonomy with the essential role of the caregiver network to ensure the safety and continuity of the hospital-level care provided at home [1].

In this context, nursing records are an essential component of medical records, as they document the patient’s status and progress, enable risk identification, and ensure continuity of care despite less frequent visits compared with hospital settings [8,9]. Accurate and complete documentation of nursing assessments helps prevent errors and improves communication among professionals [10]. However, its use in home settings is less consistent than it is in hospitals [11], largely because of structural and organisational differences between both contexts. Home care usually involves longer and more fragmented care episodes, wich may hinder both the standardization of documentation and the systematic assessment of patients’ needs and risks [12]. Furthermore, the coexistence of multiple assessment tools leads to construct overlap and redundancy, compromising the quality and validity of records [13,14]. Evidence suggests that in both hospital [15] and home settings [7,16], simplifying documentation and implementing systematic screening could ensure the collection of essential information.

The available information on integrated and validated instruments for nursing assessment in HaH is limited. Several systematic reviews highlight the lack of consensus on indicators that capture the quality of nursing care [17,18] and the scarcity of tools adapted to complex care settings, wich have been predominantly developed and studied in older populations [19]. In this regard, the Fundamentals of Care Framework [20] provides a relevant reference, particularly in its physical care dimension. This framework identifies essential care needs such as hygiene, mobility, nutrition, prevention of pressure injuries, rest, and elimination, which are interrelated with the therapeutic relationship and the care context [21,22]. Its application in HaH enables a comprehensive, person-centred nursing assessment and facilitates the identification of outcomes that are sensitive to nursing practice, such as functional decline [23], sleep disturbances [24], or malnutrition [25]. Moreover, this framework offers a robust conceptual basis for incorporating dimensions that allow the synergistic identification of risks such as falls [26], pressure injuries [27], and frailty [28].

An innovative proposal to overcome these limitations is the development of meta-instruments, understood as integrated assessment instruments that combine items and constructs from multiple validated tools into a single parsimonious instrument while maintaining clinimetric properties and diagnostic capacity equivalent to those of the original tools [29]. For instance, the VALENF Instrument [30,31] is a predictive algorithm that estimates functional capacity, pressure injury risk, and fall risk by merging the Barthel, Braden, and Downton scales into a seven-item solution. This meta-instrument has shown high predictive accuracy and reliability for the original scales’ total scores, along with adequate clinimetric properties.

Adapting this approach to hospital-at-home care could optimize nurses’ time, enhance the early detection of risks, and promote the standardization of care [16]. Therefore, it seems pertinent to develop and validate a specific meta-instrument for hospital-at-home settings aimed at supporting more parsimonious, standardized, and person-centred nursing practices. This instrument, named VALENF-HAD (from the Spanish VALoración ENFermera–Hospitalización A Domicilio, meaning “Nursing Assessment – Hospital-at-Home”), is designed to integrate multiple dimensions of nursing assessment in hospital-at-home settings, including functional capacity, pressure injury risk, fall risk, frailty, nutritional status, and sleep quality.

The overall objective of this study is to develop and validate a meta-instrument (VALENF-HAD) that integrates the assessment of functional capacity, pressure injury risk, fall risk, frailty, nutritional status, and sleep quality in patients admitted to hospital-at-home units based on the analysis of validated nursing assessment instruments. Specifically, the study aims to identify patient profiles with similar care needs based on nursing assessment, analyse the influence of sociodemographic and care-related variables on assessment outcomes, examine the relationships among the included instruments to identify redundant items, and determine the clinimetric properties of the VALENF-HAD meta-instrument, including content validity, construct validity, and internal consistency.

## Methods

### Design

**Fig 1:**
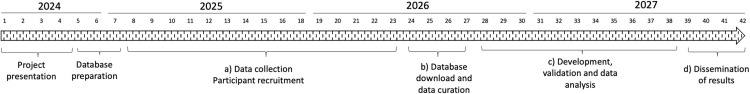
Timeline of the study.

This multicentre, cross-sectional study involves the consecutive recruitment of patients admitted to the hospital-at-home (HaH) units of four public hospitals in the province of Castellón, Spain. These hospital-at-home units are organizationally integrated within the participating hospitals and provide hospital-level care delivered in patients’ home. At the time of manuscript submission, the study is in the recruitment and data collection phase. The planned study timeline is as follows: a) participant recruitment and data collection form 01/02/2025 to 31/05/2026; b) database download and data curation from 01/06/2026 to 31/08/2026; c) data analysis from 01/09/2026 to 31/08/2027; d) dissemination of study results from 01/09/2027 onward. The overall study timeline is shown in Fig 1.

### Participants and sample

The study population will consist of nursing assessments performed on adult patients receiving Hospital-at-Home care in four public hospitals in the province of Castellón (Spain), which together provide health care coverage to 615.188 people: Hospital Universitario Comarcal de Vinaròs (HUCV), Hospital Universitario de La Plana (HULP), Consorcio Hospitalario Provincial de Castellón (CHPC), and Hospital General Universitario de Castellón (HGUCS).

The study will include nursing assessments conducted within the first 48 hours after admission for patients aged 18 years or older who agree to participate and provide written informed consent, as the assessment instruments included in the study are primarily validated in adult populations. The first hours were selected because they correspond to the initial nursing assessment period in hospital-at-home units, allowing for a standardized baseline evaluation. Assessments from patients whose expected stay is less than 48 hours, such as those admitted under a day hospital regimen, will be excluded.

Regarding sample size, the literature recommends including between 5 and 10 participants per item in studies aimed at the development and validation of questionnaires [32]. The total number of items across all the instruments considered in this study is 59; therefore, a minimum of 590 assessments was initially estimated. However, due to the lack of specific recommendations for studies involving the integration of multiple assessment instruments and based on previous methodological references [29], the sample size was increased to 1.180 assessments to ensure adequate representativeness and to support more complex analytical strategies.

The sample will be stratified according to the number of admissions in each HaH unit, excluding 20% corresponding to day-hospital admissions. Consecutive sampling will be performed in each until the required sample size is reached, as shown in Table 1.

**Table 1:** Sample size estimation.

Hospital	Admissions in 2022	Average monthly admissions	Estimated n (12 months)
HUCV	1.136	94	318
HULP	1.105	92	311
CHPC	889	74	251
HGUCS	1.066	88	300
**TOTAL**	4.196	348	**1.180**

### Variables and instruments

Sociodemographic variables are included (age, sex, educational level, marital status, type of cohabitation, presence of a caregiver, type of caregiver, and place where care is provided); variables related to the care process (hospital, source of admission, type of process, clinical profile, advanced or terminal palliative situation, Charlson Comorbidity Index [33], active infection on admission, pressure injury on admission, bedridden status, presence of invasive clinical devices, polypharmacy, pain, and presence of symptoms assessed using the Edmonton Symptom Assessment System [34]; and palliative sedation status, which is measured with the RAMSAY Sedation Scale [35].

Variables related to the nursing assessment will be collected, including functional capacity, measured with the Barthel Index [36] and the Lawton and Brody scale [37]; pressure injury risk, measured with the Braden Index [38]; and fall risk, measured with the STRATIFY scale [39]. These assessments will be recorded in the data collection form together with the VALENF Instrument [30], a meta-instrument that integrates the assessment of functional capacity, pressure injury risk, and fall risk. In addition, frailty will be measured with the FRAIL questionnaire [40], nutritional status with the NSI tool [41], and sleep quality with the Athens Insomnia Scale [42].

### Data collection

Data collection will be conducted prospectively over an estimated 16-month period (01/02/2025 to 31/05/2026) through consecutive sampling in the home hospitalization units participating in the study. The time required for a complete evaluation in routine clinical practice is approximately 20 minutes. Data will be collected within the first 48 hours after admission, allowing for an initial assessment on the first day followed by a more comprehensive evaluation on the second. This phased approach has been designed to reduce the pressure of the first visit and minimize potential fatigue among both patients and clinical staff. Furthermore, the HaH model allows for longer and more flexible interaction with patients compared to conventional hospitalization. Data entry will be carried out using Research Electronic Data Capture (REDCap) software.

A common Standard Operating Procedure (SOP) was developed for all participating centers, detailing the instructions for instrument administration and data recording. Prior to the start of data collection, structured training sessions were conducted at each participating hospital to ensure consistency in the application of the instruments by nursing professionals. During the data collection period, continuous monitoring mechanisms were established to ensure measurement consistency, including regular communication between the research team and clinical staff, as well as periodic data quality checks to identify potential inconsistencies or deviations in data collection.

After informing patients and providing them with the study information sheet, written informed consent will be obtained. Decision-making capacity will be clinically assessed by the nursing team as part of routine care, based on the patients’s ability to understand the study information and provide informed consent. In cases where patients present cognitive impairment or are unable to provide valid consent, informed consent will be obtained from a legally authorized representative (LAR), in accordance with standard ethical procedures.

When necessary, the primary caregiver and/or family member will complete the questionnaires as proxy respondents. Proxy responses will be explicity identified in the dataset, allowing them to be distinguished from patient self-reported data.

Both the nursing professionals and the research team signed a confidentiality and data responsibility agreement, as well as an intellectual property commitment.

### Statistical analysis

The statistical analysis will be conducted in four sequential phases. First, the sample and measurement instruments will be characterized through a descriptive analysis according to the nature of the variables. Significant differences in the assessment results of problems related to hospitalization will be examined, considering sociodemographic and care process variables, taking into account whether the data are patient self-reported or proxy-reported. The normality and homoscedasticity of the sample will be tested using the Kolmogorov–Smirnov and Levene tests, respectively. Depending on the results, Student’s t test or ANOVA will be applied, depending on the number of groups. Categorical variables will be analysed using the chi-square (χ²) test, and correlations will be assessed using Pearson’s or Spearman’s test, as appropriate. When the assumptions for these tests are not met, the corresponding nonparametric tests will be applied.

Following this initial analysis, the development and validation process of the new meta-instrument will be carried out by adapting the methodological proposal of Llagostera-Reverter et al. [29]. First, an analysis will be performed to explore the conceptual and semantic relationships among the dimensions of the instruments. In addition, the items of the instruments will be analysed to identify similarities, duplications, and redundancies, with the goal of establishing direct or indirect conceptual relationships through a nominal group [43] composed of members of the research team and nursing professionals working in home hospitalization units and analysing nursing physical care within the conceptual framework of the Fundamentals of Care [44]. Subsequently, conceptual similarities and redundancies between the dimensions and items of the instruments will be examined, and correlations will be tested using Pearson’s or Spearman’s tests, as appropriate. Multiple correspondence analysis and cluster analysis will be used to explore potential groupings of items and to identify clinical profiles of patients with similar care needs [45–47].

Second, linear model analyses will be performed using each assessment instrument as a dependent variable to determine the influence of sociodemographic and care process variables. Additional linear model analyses will be conducted to predict the scores of the original instruments on the basis of combinations of their own items.

Third, the initial validation of the new meta-instrument will be conducted. Agreement will be assessed using the intraclass correlation coefficient (ICC > 0.7) [48]. Participants will also be grouped according to the categorical levels of each assessment instrument on the basis of their obtained scores, and overall and category-specific agreement will be examined using Kendall’s tau-b and Cohen’s kappa tests (τ-b and κ > 0.7) [49]. To ensure the robustness and generalizability of these predictive models, cross-validation will be applied [50].

Fourth, the clinimetric properties will be determined following the recommendations of the COSMIN initiative [51]. Content validity will be evaluated through a nominal group of clinical nurses with at least 10 years of experience and university nursing faculty with doctoral degrees. They will assess the relevance of the proposed items and dimensions using the Polit and Beck (2006) methodology to calculate the item-level content validity index (I-CVI ≥ 0.78) and the scale-level content validity index (S-CVI ≥ 0.9) [52]. Construct validity will be assessed according to the methodology proposed by Efron and Tibshirani [53]. The sample will be randomly divided into two subgroups. In the first subgroup, an exploratory factor analysis with oblimin rotation and maximum likelihood estimation will be performed after verifying adequacy through Kaiser–Meyer–Olkin and Bartlett’s sphericity tests. In the second subgroup, confirmatory factor analysis and structural equation modelling will be conducted to validate the theoretical structure of the instrument. Model fit will be evaluated using χ²/df (<5), RMSEA (<0.05), and CFI (≥0.97). Internal consistency will be estimated using McDonald’s Omega (Ω > 0.7) [54] and Cronbach’s alpha (α > 0.7), as appropriate, depending on the characteristics of the data and the underlying assumptions of each method [55]. Finally, interrater reliability will be assessed in a pilot sample using the intraclass correlation coefficient (ICC > 0.61). A p value < 0.05 will be considered to indicate statistical significance, and analyses will be conducted using software, selected according to their specific analytical capabilities for descriptive, multivariate, and advanced modelling procedures.

The Statistical Analysis Plan (SAP) was defined during the study design phase, prior to the start of participant recruitment. This plan remained stable throughout the study and was considered finalized before the initiation of data analysis. The study was retrospectively registered (ISRCTN 94528203, 09/05/2025), including the objectives, variables, and analytical strategies. No relevant changes were made after the start of recruitment.

### Data management plans

All data will be collected using the Research Electronic Data Capture (REDCap) platform, hosted on secure institutional servers at the Universitat Jaume I. Access to the database will be password-protected and restricted to authorized members of the research team. Each participant will be assigned a unique identification code to ensure pseudonymization, and no personal identifiers will be stored in the analytical dataset.

Data entry will be validated through automated range and logic checks to minimize transcription errors. Weekly quality control procedures will be conducted by the principal investigator to detect and correct missing or inconsistent entries. The final dataset will be stored in encrypted format and backed up regularly in compliance with institutional and European data protection regulations (Regulation [EU] 2016/679 and Spanish Organic Law 3/2018).

No datasets were generated or analysed during the current study. Upon completion of the study, the anonymized dataset at item level will be made publicly available in the institutional repository of Universitat Jaume I (UJI). All data will be processed in accordance with applicable data protection regulations. Prior to data sharing, all directly and indirectly identifiable information will be removed to ensure full anonymization and protect participant confidentiality.

### Ethical considerations

The project received positive evaluations from the Research and Ethics Committees of the participating hospitals (HGUC [version 2, code VALENF-HAD], HULP [code VALENF-HAD], and CHPC [minutes no. 65, code VALENF-HAD]). The Hospital Universitario Comarcal de Vinaròs (HUCV) did not have an independent ethics committee at the time of study initiation. However, its participation was conducted under the same study protocol previously approved by the aforementioned Research Ethics Committees, ensuring ethical consistency across all sites. Institutional authorization was additionally obtained from the HUCV management board in accordance with local procedures. The Hospital General Universitario de Castellón (HGUC) acted as the reference centre for ethical approval within the multicentre study framework. This study was designed in accordance with Spanish Organic Law 3/2018 of December 5 on Personal Data Protection and Guarantee of Digital Rights, as well as with Regulation (EU) 2016/679 of the European Parliament and of the Council of 27 April 2016 on the protection of natural persons.

Participants will be clearly informed, at the appropriate time, about the purpose and procedures of the study as well as their rights to access information related to their participation and to withdraw from the study at any time if they wish. This information will be provided by the nursing team involved in the study. After receiving this information, participants will be given the informed consent form to voluntarily sign. The database will not contain any personal information that could be used to identify participants.

## Discussion

The application of meta-instruments in nursing assessment offers significant advantages through the integration of common constructs from different instruments into a single tool, thereby reducing redundancies and simplifying documentation while promoting the collection of higher-quality information. Furthermore, streamlining the completion process allows nurses to devote more time to direct patient care and enhances the detection of risk situations, facilitating clinical decision-making.

However, the literature on how to develop and validate meta-instruments remains scarce [29], and few previous studies have been reported, all of which employed retrospective cross-sectional designs [30,56]. In fact, we did not identify any study with a similar aim that did not use registry data. The choice of a prospective design in this study is based on the variability in the assessment systems used across the four participating home hospitalization units. Indeed, a necessary preliminary step was reaching a consensus on the assessment dimensions and measurement instruments to be used.

In addition, previous studies on meta-instruments such as the VALENF instrument have demonstrated a strong capacity to detect clinically relevant events, including functional decline, risk of falls, and pressure injuries. These findings support the potential utility of integrated assessment tools not only to simplify documentation but also to enhance early risk identification and clinical decision-making [57].

This study design is intended to help minimize the information bias associated with the quality of data recorded in clinical histories [58]. Nonetheless, one of the main challenges of this study is maintaining the motivation of the nursing staff to collaborate over at least a 12-month data collection period and achieving the required sample size. To address these limitations, comprehensive training sessions on the REDCap platform were provided to professionals in each home hospitalization unit prior to data collection to ensure standardized measurements. In addition, during data collection, the research team maintains regular contact with the clinical nursing teams to resolve questions and issues related to recruitment and data collection. The researchers, together with the designated lead in each home hospitalization unit, perform weekly monitoring in the CDRe system to identify potential missing or erroneous data.

## Supporting information

S1 File SPIRIT Checklist. (DOCX)

## References

[1] Domínguez BM. La hospitalización a domicilio en el siglo XXI. Hosp Domic. 2017;1:7–9. doi: 10.22585/hospdomic.v1i1.8

[2] Diaz-Gegundez M, Gomez de Argila I, Ferrer-Cobo E, Castelar-Delgado E, Diaz-Gegundez M, Gomez de Argila I. 10 años de hospitalización a domicilio en el entorno de un hospital comarcal. Hosp Domic. 2020;4:69–80. doi: 10.22585/hospdomic.v4i2.96

[3] Song J, Zolnoori M, McDonald MV, Barrón Y, Cato K, Sockolow P, et al. Factors associated with timing of the start-of-care nursing visits in home health care. J Am Med Dir Assoc. 2021;22(11):2358-2365.e3. doi: 10.1016/j.jamda.2021.03.005 33844990 PMC8501154

[4] Ouchi K, Liu S, Tonellato D, Keschner YG, Kennedy M, Levine DM. Home hospital as a disposition for older adults from the emergency department: Benefits and opportunities. J Am Coll Emerg Physicians Open. 2021;2(4):e12517. doi: 10.1002/emp2.12517 34322684 PMC8295243

[5] Hobensack M, Song J, Scharp D, Bowles KH, Topaz M. Machine learning applied to electronic health record data in home healthcare: A scoping review. Int J Med Inform. 2023;170:104978. doi: 10.1016/j.ijmedinf.2022.104978 36592572 PMC9869861

[6] Lee J, Kang M-J, Garcia JP, Dykes PC. Developing hierarchical standardized home care nursing statements using nursing standard terminologies. Int J Med Inform. 2020;141:104227. doi: 10.1016/j.ijmedinf.2020.104227 32763790

[7] Flyum IR, Gjevjon ER, Eklund AJ, Borglin G. What do we know about nursing practice in relation to functional ability limitations, frailty and models of care among older people in home- and facility-based care: a scoping review. BMC Nurs. 2025;24(1):406. doi: 10.1186/s12912-025-02948-7 40211311 PMC11987274

[8] Slyngstad L, Helgheim BI. How do different health record systems affect home health care? a cross-sectional study of electronic- versus manual documentation system. Int J Gen Med. 2022;15:1945–56. doi: 10.2147/IJGM.S346366 35237067 PMC8882660

[9] Gungor S, Akcoban S. Change in care dependency and quality of home care nursing assessment levels following home health services in patients receiving home health care. Geriatr Nurs. 2025;65:103456. doi: 10.1016/j.gerinurse.2025.103456 40628103

[10] Soza Diaz CDF, Bazán Sánchez ACL, Diaz Manchay RJ, Soza Diaz CDF, Bazán Sánchez ACL, Diaz Manchay RJ. Percepción de las enfermeras sobre el uso de sus registros para garantizar la continuidad del cuidado. 2020;14.

[11] Hertzum M. Electronic health records in danish home care and nursing homes: inadequate documentation of care, medication, and consent. Appl Clin Inform. 2021;12(1):27–33. doi: 10.1055/s-0040-1721013 33440430 PMC7806422

[12] Bjerkan J, Valderaune V, Olsen RM. Patient safety through nursing documentation: Barriers identified by healthcare professionals and students. Frontiers in Computer Science. 2021;3. doi: 10.3389/fcomp.2021.624555

[13] Brown JA, Cooper AL, Albrecht MA. Development and content validation of the Burden of Documentation for Nurses and Midwives (BurDoNsaM) survey. J Adv Nurs. 2020;76(5):1273–81. doi: 10.1111/jan.14320 32027387

[14] Iula A, Ialungo C, de Waure C, Raponi M, Burgazzoli M, Zega M, et al. Quality of care: ecological study for the evaluation of completeness and accuracy in nursing assessment. Int J Environ Res Public Health. 2020;17(9):3259. doi: 10.3390/ijerph17093259 32392838 PMC7246491

[15] Jacques D, Will J, Dauterman D, Zavotsky KE, Delmore B, Doty GR, et al. Evaluating nurses’ perceptions of documentation in the electronic health record: multimethod analysis. JMIR Nurs. 2025;8:e69651. doi: 10.2196/69651 40294588 PMC12052386

[16] Larjow E, von Fintel M, Busse A. A mixed-methods study of quality differences between applied documentation approaches in nursing homes. BMC Nurs. 2022;21(1):265. doi: 10.1186/s12912-022-01046-2 36171628 PMC9520897

[17] Oner B, Zengul FD, Oner N, Ivankova NV, Karadag A, Patrician PA. Nursing-sensitive indicators for nursing care: a systematic review (1997-2017). Nurs Open. 2021;8(3):1005–22. doi: 10.1002/nop2.654 34482649 PMC8046086

[18] Gormley E, Connolly M, Ryder M. The development of nursing-sensitive indicators: a critical discussion. Int J Nurs Stud Adv. 2024;7:100227. doi: 10.1016/j.ijnsa.2024.100227 39188551 PMC11345314

[19] Fang W, Phung H, Olley R, Lee P. The major domains of comprehensive assessment tools for older adults requiring home-based aged care services: a systematic review. Healthcare (Basel). 2024;12(23):2468. doi: 10.3390/healthcare12232468 39685089 PMC11641082

[20] Kitson A, Wiechula R, Conroy T, Muntlin A A, Whitaker N. The future shape of the nursing workforce: a synthesis of the evidence of factors that impact on quality nursing care. Int J Evidence-Based Healthcare. 2013;11:228. doi: 10.1097/01258363-201309000-00038

[21] Feo R, Conroy T, Jangland E, Muntlin Athlin Å, Brovall M, Parr J, et al. Towards a standardised definition for fundamental care: a modified Delphi study. J Clin Nurs. 2018;27(11–12):2285–99. doi: 10.1111/jocn.14247 29278437

[22] Zwakhalen SMG, Hamers JPH, Metzelthin SF, Ettema R, Heinen M, de Man-Van Ginkel JM, et al. Basic nursing care: the most provided, the least evidence based - A discussion paper. J Clin Nurs. 2018;27(11–12):2496–505. doi: 10.1111/jocn.14296 29399942

[23] Hoogerduijn JG, Buurman BM, Korevaar JC, Grobbee DE, de Rooij SE, Schuurmans MJ. The prediction of functional decline in older hospitalised patients. Age Ageing. 2012;41(3):381–7. doi: 10.1093/ageing/afs015 22378613

[24] Carrera-Hernández L, Aizpitarte-Pejenaute E, Zugazagoitia-Ciarrusta N, Goñi-Viguria R. Percepción del sueño de los pacientes en una Unidad de Cuidados Intensivos. Enferm Intensiva. 2018;29:53–63. doi: 10.1016/j.enfi.2018.01.002 29605589

[25] Castro-Vega I, Veses Martín S, Cantero Llorca J, Barrios Marta C, Bañuls C, Hernández-Mijares A. Validity, efficacy and reliability of 3 nutritional screening tools regarding the nutritional assessment in different social and health areas. Med Clin (Barc). 2018;150(5):185–7. doi: 10.1016/j.medcli.2017.07.019 28947299

[26] Riaño Castañeda MG, Moreno Gómez J, Echeverría Avellaneda LS, Rangel Caballero LG, Sánchez Delgado JC. Condición física funcional y riesgo de caídas en adultos mayores. Rev Cuba Investig Bioméd. 2018;37:1–10.

[27] Kaşıkçı M, Aksoy M, Ay E. Investigation of the prevalence of pressure ulcers and patient-related risk factors in hospitals in the province of Erzurum: A cross-sectional study. J Tissue Viability. 2018;27(3):135–40. doi: 10.1016/j.jtv.2018.05.001 29776817

[28] Acosta-Benito MÁ, Martín-Lesende I. Fragilidad en atención primaria: diagnóstico y manejo multidisciplinar. Atención Primaria. 2022;54(9):102395. doi: 10.1016/j.aprim.2022.102395 35700618 PMC9198324

[29] Llagostera-Reverter I, Luna-Aleixós D, Valero-Chillerón MJ, González-Chordá VM. Desarrollo y validación de meta-instrumentos de medición: una aproximación metodológica. Enfermería Clínica. 2024;34(4):322–9. doi: 10.1016/j.enfcli.2024.04.002 39067617

[30] Luna-Aleixos D, Llagostera-Reverter I, Castelló-Benavent X, Aquilué-Ballarín M, Mecho-Montoliu G, Cervera-Gasch Á, et al. Development and Validation of a Meta-Instrument for Nursing Assessment in Adult Hospitalization Units (VALENF Instrument) (Part I). Int J Environ Res Public Health. 2022;19(22):14622. doi: 10.3390/ijerph192214622 36429341 PMC9690557

[31] Luna-Aleixos D, Llagostera-Reverter I, Castelló-Benavent X, Aquilué-Ballarín M, Mecho-Montoliu G, Cervera-Gasch Á, et al. Development and Validation of a Meta-Instrument for the Assessment of Functional Capacity, the Risk of Falls and Pressure Injuries in Adult Hospitalization Units (VALENF Instrument) (Part II). Int J Environ Res Public Health. 2023;20(6):5003. doi: 10.3390/ijerph20065003 36981915 PMC10049057

[32] Anthoine E, Moret L, Regnault A, Sébille V, Hardouin J-B. Sample size used to validate a scale: a review of publications on newly-developed patient reported outcomes measures. Health Qual Life Outcomes. 2014;12:176. doi: 10.1186/s12955-014-0176-2 25492701 PMC4275948

[33] Rosas-Carrasco O, González-Flores E, Brito-Carrera AM, Vázquez-Valdez OE, Peschard-Sáenz E, Gutiérrez-Robledo LM. Evaluación de la comorbilidad en el adulto mayor. Rev Médica Inst Mex Seguro Soc. 2011;49:153–62. 21703142

[34] Carvajal Valcárcel A, Martínez García M, Centeno Cortés C. Versión española del Edmonton Symptom Assessment Sytem (ESAS): un instrumento de referencia para la valoración sintomática del paciente con cáncer avanzado. Medicina Paliativa. 2013;20(4):143–9. doi: 10.1016/j.medipa.2013.02.001

[35] Frade Mera MJ, Guirao Moya A, Esteban Sánchez ME, Rivera Álvarez J, Cruz Ramos AM, Bretones Chorro B. Análisis de 4 escalas de valoración de la sedación en el paciente crítico. Enferm Intensiva. 2009;20:88–94. doi: 10.1016/S1130-2399(09)72588-X 19775565

[36] González DC, Caicoya AM, Sánchez AF, García VA, González JVG, Palacios ED. Evaluación de la fiabilidad y validez de una escala de valoración social en el anciano. Aten Primaria Publ Of Soc Esp Fam Comunitaria. 1999;23:434–40. 10363397

[37] Vergara I, Bilbao A, Orive M, Garcia-Gutierrez S, Navarro G, Quintana JM. Validation of the Spanish version of the Lawton IADL Scale for its application in elderly people. Health Qual Life Outcomes. 2012;10:130. doi: 10.1186/1477-7525-10-130 23110491 PMC3541128

[38] Patricia Moreno-Pina J, Richart-Martínez M, Adolf Guirao-Goris J, Duarte-Climents G. Análisis de las escalas de valoración del riesgo de desarrollar una úlcera por presión. Enfermería Clínica. 2007;17(4):186–97. doi: 10.1016/s1130-8621(07)71795-3 17915121

[39] Aranda-Gallardo M, Enriquez de Luna-Rodriguez M, Vazquez-Blanco MJ, Canca-Sanchez JC, Moya-Suarez AB, Morales-Asencio JM. Diagnostic validity of the STRATIFY and Downton instruments for evaluating the risk of falls by hospitalised acute-care patients: a multicentre longitudinal study. BMC Health Serv Res. 2017;17(1):277. doi: 10.1186/s12913-017-2214-3 28412939 PMC5393002

[40] Rosas-Carrasco O, Cruz-Arenas E, Parra-Rodríguez L, García-González AI, Contreras-González LH, Szlejf C. Cross-cultural adaptation and validation of the FRAIL scale to assess frailty in Mexican adults. J Am Med Dir Assoc. 2016;17(12):1094–8. doi: 10.1016/j.jamda.2016.07.008 27567463

[41] Mitchell DC, Smiciklas-Wright H, Friedmann JM, Jensen G. Dietary intake assessed by the Nutrition Screening Initiative Level II Screen is a sensitive but not a specific indicator of nutrition risk in older adults. J Am Diet Assoc. 2002;102(6):842–4. doi: 10.1016/s0002-8223(02)90188-4 12067053

[42] Gómez-Benito J, Ruiz C, Guilera G. A Spanish version of the Athens Insomnia Scale. Qual Life Res. 2011;20(6):931–7. doi: 10.1007/s11136-010-9827-x 21210225

[43] Bhandari S, Hallowell MR. Identifying and controlling biases in expert-opinion research: Guidelines for variations of Delphi, nominal group technique, and focus groups. J Manag Eng. 2021;37:04021015. doi: 10.1061/(ASCE)ME.1943-5479.0000909

[44] Kitson AL, Muntlin Athlin A, Conroy T. Anything but basic: nursing’s challenge in meeting patients’ fundamental care needs. J Nurs Scholarsh. 2014;46(5):331–9. doi: 10.1111/jnu.12081 24758508

[45] Botero VHC, Cuervo EC, Bossio MC. Acoso en el ambiente escolar: análisis de un cuestionario mediante teoría de respuesta al ítem y análisis de correspondencias múltiples. Univ Psychol. 2014;13:443–56.

[46] Barth A. The changing nature of attitude constructs: an application of multiple correspondence analysis on gender role attitudes. Qual Quant. 2016;50:1507–23. doi: 10.1007/s11135-015-0218-9

[47] Guinot C, Latreille J, Malvy D, Preziosi P, Galan P, Hercberg S, et al. Use of multiple correspondence analysis and cluster analysis to study dietary behaviour: food consumption questionnaire in the SU.VI.MAX. cohort. Eur J Epidemiol. 2001;17(6):505–16. doi: 10.1023/a:1014586129113 11949721

[48] Koo TK, Li MY. A guideline of selecting and reporting intraclass correlation coefficients for reliability research. J Chiropr Med. 2016;15(2):155–63. doi: 10.1016/j.jcm.2016.02.012 27330520 PMC4913118

[49] de Raadt A, Warrens MJ, Bosker RJ, Kiers HAL. A comparison of reliability coefficients for ordinal rating scales. J Classif. 2021;38:519–43. doi: 10.1007/s00357-021-09386-5

[50] Moons KGM, Altman DG, Reitsma JB, Ioannidis JPA, Macaskill P, Steyerberg EW, et al. Transparent Reporting of a multivariable prediction model for Individual Prognosis or Diagnosis (TRIPOD): explanation and elaboration. Ann Intern Med. 2015;162(1):W1-73. doi: 10.7326/M14-0698 25560730

[51] COSMIN. Checklists for Assessing Study Qualities. [cited 2025 July 11]. https://www.cosmin.nl/tools/checklists-assessing-methodological-study-qualities/

[52] Polit DF, Beck CT. The content validity index: are you sure you know what’s being reported? Critique and recommendations. Res Nurs Health. 2006;29(5):489–97. doi: 10.1002/nur.20147 16977646

[53] Efron B, Tibshirani R. Improvements on cross-validation: the 632 bootstrap method. J Am Stat Assoc. 1997;92:548–60. doi: 10.1080/01621459.1997.10474007

[54] Kalkbrenner MT. Alpha, omega, and h internal consistency reliability estimates: reviewing these options and when to use them. Couns Outcome Res Eval. 2023;14:77–88.

[55] Ponterotto JG, Ruckdeschel DE. An overview of coefficient alpha and a reliability matrix for estimating adequacy of internal consistency coefficients with psychological research measures. Percept Mot Skills. 2007;105(3 Pt 1):997–1014. doi: 10.2466/pms.105.3.997-1014 18229554

[56] Palese A, Marini E, Guarnier A, Barelli P, Zambiasi P, Allegrini E, et al. Overcoming redundancies in bedside nursing assessments by validating a parsimonious meta-tool: findings from a methodological exercise study. J Eval Clin Pract. 2016;22(5):771–80. doi: 10.1111/jep.12539 27144880

[57] Luna-Aleixos D, González-Chordá VM, Ortíz-Mallasén V, Llagostera-Reverter I, Machancoses FH, Cervera-Gasch Á, et al. VALENF-instrument-based nursing assessment and early occurrence of hospital-acquired pressure injuries and falls among hospitalized adults. Nurs Rep. 2026;16(3):80. doi: 10.3390/nursrep16030080 41893068 PMC13028764

[58] Goldstein ND. Electronic health records in epidemiology: appropriate questions, common biases, and potential sensitivity analyses. Curr Epidemiol Rep. 2025;12:11. doi: 10.1007/s40471-025-00365-7

